# Evaluation of Equalization of Cancer Medical Services in Japan Using Public Database in Japan

**DOI:** 10.2188/jea.JE20180057

**Published:** 2018-11-05

**Authors:** Tomotaka Sobue

**Affiliations:** Division of Environmental Medicine and Population Sciences, Department of Social and Environmental Medicine, Graduate School of Medicine, Osaka University, Osaka, Japan

In Japan, the first basic plan for promoting cancer control programs was developed and started in 2007. Since then, equalization of cancer medical services is one of the important issues in cancer control in this country.

In this issue, Tanaka et al reported the distribution of travel time to hospital among cancer patients based on individual data from the Patients Survey and the Survey of Medical Institutions conducted in January 2011.^1^ They used data from 50,845 cancer inpatients and analyzed the geographical information system (GIS)-derived cancer patients’ travel time for hospital admission. It was shown that patients with stomach or colorectal cancer tended to admit to nearby hospitals compared with those with cervical cancer or leukemia. The travel time tended to be longer for younger patients and patients living in the secondary healthcare service areas without designated cancer care hospitals. They also reported that there is inequity of travel time between prefectures.

Travel time is one of the factors to be considered when promoting equalization of cancer medical services. Since the actual data in this field is substantially lacking, the article reported by Tanaka et al is valuable, especially at the national level. The major drawback of their article, however, is that they could use only broad address information (city, town, and village), not detailed information of each cancer patient. This can be supplemented by information from another database, such as health insurance claims data.^2^

Another factor to be considered in the process of equalization is quality of cancer care. In order to improve the situation of long travel time, allocating additional hospitals may be the solution. However, it may not be good, not only because additional cost is needed, but also because fewer patients per hospitals may worsen the quality of cancer care in each hospital. Data on the quality of cancer care has been collected among designated cancer care hospitals using the hospital-based cancer registry system.

As a next step, studies considering both travel time and quality of cancer care will be needed.
